# Identification of MX2 as a Novel Prognostic Biomarker for Sunitinib Resistance in Clear Cell Renal Cell Carcinoma

**DOI:** 10.3389/fgene.2021.680369

**Published:** 2021-07-09

**Authors:** Yuang Wei, Xinglin Chen, Xiaohan Ren, Bao Wang, Qian Zhang, Hengtao Bu, Jian Qian, Pengfei Shao

**Affiliations:** Department of Urology, The First Affiliated Hospital of Nanjing Medical University, Nanjing, China

**Keywords:** sunitinib resistance, drug resistance, antiangiogenic therapy, TKIs, clear cell renal cell carcinoma, human myxovirus resistance protein 2, Prognosis

## Abstract

**Background:**

Antiangiogenic agents that specifically target vascular endothelial growth factor receptor (VEGFR), such as sunitinib, have been utilized as the standard therapy for metastatic clear cell renal cell carcinoma (ccRCC) patients. However, most patients eventually show no responses to the targeted drugs, and the mechanisms for the resistance remain unclear. This study is aimed to identify pivotal molecules and to uncover their potential functions involved in this adverse event in ccRCC treatment.

**Methods:**

Two datasets, GSE64052 and GSE76068, were obtained from the Gene Expression Omnibus (GEO) database. The differentially expressed genes (DEGs) were identified using the limma package in R software. The gene set enrichment analysis (GSEA) was conducted using clusterProfiler package. A protein–protein interaction (PPI) network was built using the STRING database and Cytoscape software. Kaplan—Meier survival curves were plotted using R software. qRT-PCR and Western blotting were used to detect the MX2 and pathway expression in RCC cell lines. Sunitinib-resistant cell lines were constructed, and loss-of-function experiments were conducted by knocking down MX2. All statistical analyses were performed using R version 3.6.1 and SPSS 23.0.

**Results:**

A total of 760 DEGs were derived from two datasets in GEO database, and five hub genes were identified, among which high-level MX2 exhibited a pronounced correlation with poor overall survival (OS) in sunitinib-resistant ccRCC patients. Clinical correlation analysis and Gene Set Variation Analysis (GSVA) on MX2 showed that the upregulation of MX2 was significantly related to the malignant phenotype of ccRCC, and it was involved in several pathways and biological processes associated with anticancer drug resistance. qRT-PCR and Western blotting revealed that MX2 was distinctly upregulated in sunitinib-resistant RCC cell lines. Colony formation assay and Cell Counting Kit-8 (CCK8) assay showed that MX2 strongly promoted resistant capability to sunitinib of ccRCC cells.

**Conclusion:**

MX2 is a potent indicator for sunitinib resistance and a therapeutic target in ccRCC patients.

## Introduction

Kidney cancer is a lethal urological disease and one of the most malignant tumors in human beings. As estimated, kidney cancer will account for up to 73,700 new cases and 14,800 deaths in the United States in 2020 (Siegel et al., 2020). Renal cell carcinoma (RCC) is commonly divided into three main different histological subtypes, among which clear cell RCC (ccRCC) is the most frequent with a proportion of 75–80% (Motzer et al., 1996). Over 90% of ccRCC has sporadic mutations of the von Hippel-Lindau (VHL) gene located on human chromosome 3 p, which leads to the excessive vascularization of tumor tissues (Jonasch et al., 2014). Normally, localized carcinoma can be treated with active surveillance and partial/radical nephrectomy, while patients with metastatic ccRCC require systematic treatment to obtain a better survival (Curti, 2004). With in-depth knowledge of the pathophysiology of ccRCC, the advent of therapeutic agents targeting the vascular endothelial growth factor (VEGF) signaling axis has become a milestone in ccRCC therapy (Rini et al., 2009). Notably, multitargeted tyrosine kinase inhibitors (TKIs), represented by sunitinib, have shown decent efficacy on metastatic RCC (mRCC) (Motzer et al., 2013).

Today, there are several Food and Drug Administration (FDA)-approved therapies of ccRCC for first-line and second-line standard treatments targeting a wide range of targets, including VEGF (bevacizumab), VEGFR/PDGFR (lenvatinib, cabozantinib, pazopanib, axitinib, sorafenib, and sunitinib), mTOR (everolimus and temsirolimus), PD-1 (nivolumab and pembrolizumab), and PD-L1 (avelumab and atezolizumab) (Yang and Chen, 2020). Despite the effectiveness of targeted therapy on metastatic ccRCC, many patients would eventually develop resistance to the antiangiogenic therapy with a median time of 6–15 months, followed by poor overall survival (OS) (Motzer et al., 2007; Bergers and Hanahan, 2008; Molina et al., 2014). Although the advent of immunotherapy with checkpoint inhibitors and sequential use of targeted agents has brought a promising treatment landscape for advanced ccRCC, it is urgent to elucidate the precise mechanisms of resistance to VEGFR-TKIs (Rini and Atkins, 2009).

In this study, we introduced a key molecule with aberrant expression in sunitinib-resistant ccRCC. Human myxovirus resistance protein 2 (MX2, also designated MXB), located on chromosome 21q22.3, is a member of the GTPase family (Haller et al., 2015). MX2 is mainly induced by interferon-alpha (INF-α) through potent antiviral activity against HIV-1 (Goujon et al., 2013). Melissa and colleagues also found that depletion of MX2 significantly reduces the anti-HIV-1 potency of IFN-α, thus confirming that MX2 serves as the effector of the anti-HIV-1 activity of IFN-α (Kane et al., 2013). However, in addition to being reported as a tumor suppressor in glioblastoma (Wang et al., 2019) and melanoma (Choi et al., 2020), lately, the function of MX2 in tumors and anticancer drug-related resistance has been rarely discussed.

Here, through comprehensive bioinformatics analysis, we acquired gene expression data of ccRCC samples either sensitive or resistant to sunitinib. Then we screened for hub genes and particularly explored the biofunction of MX2. The result showed that the MX2 level was significantly higher in ccRCC compared with normal or adjacent tissues. High expression of MX2 was also associated with higher clinical stage and grade, as well as worse OS of patients. Furthermore, we found that MX2 could be involved in some TKI resistance-associated pathways and promoted the formation of sunitinib resistance in ccRCC cell lines. *In vitro* experiments were also conducted to verify these findings. It is the first time that MX2 is reported to be associated with sunitinib resistance in ccRCC, which we believe could bring new insights into the mechanisms of resistance to antiangiogenic therapies.

## Materials and Methods

### Data Acquisition and Preprocessing

The expression profile and corresponding clinical information were obtained from the Gene Expression Omnibus (GEO) and The Cancer Genome Atlas (TCGA) database. The dataset GSE64052 contained 28 ccRCC samples of patient-derived mouse xenografts (PDX), whose platform was GPL570. Among them, five samples (GSM15636509–GSM1563513) were sunitinib-sensitive, and four samples were sunitinib-resistant (GSM1563514–GSM1563517). The dataset GSE76068 also used the PDX model to identify gene expression changes during sunitinib resistance development, whose platforms were GPL6885 and GPL10558. Eight paired samples in GPL6885 were selected in our analysis for the human expression profile (GSM1973621–GSM1973636). All the data were preprocessed before the analysis beginning. In detail, the preprocessing flowchart included probe annotation, missing expression data imputation, normalization, and background correction.

### Identification of Differentially Expressed Genes and Enrichment Analysis

The differentially expressed genes (DEGs) were identified using the limma package in R software with the threshold of logFC | (fold-change)| > 1 and *p* value < 0.05. The gene set enrichment analysis (GSEA) was conducted using clusterProfiler package. The gmt file C1–C8 and hallmark were downloaded from the GSEA website as the reference gene set. Only terms with *p* value < 0.05 were selected. Gene Set Variation Analysis (GSVA) was performed to quantify the normalized enrichment score of the Hallmark pathway in high and low MX2 subtypes.

### Protein–Protein Interaction Network Construction

A PPI network was constructed using the STRING^1^ (Search Tool for the Retrieval of Interacting Genes) database, an online biological database that could be conducive to uncover the critical regulatory genes. The Cytoscape software was following used for visualization. Cytohubba, a plug-in of Cytoscape, was used to identify hub nodes according to the maximal clique centrality (MCC) value.

### Clinical Correlation Analysis

Patients with complete survival days and status from the Kidney Renal Clear Cell Carcinoma (KIRC), International Cancer Genome Consortium (ICGC), and dataset GSE29609 were selected for prognosis analysis. Kaplan–Meier survival curves were plotted using the survival package in R software to visualize patients’ prognosis differences in different groups. Clinical features of each KIRC patient were collated with the author’s own Perl node.

### Establishment of Sunitinib-Resistant Cell Lines and Cell Counting Kit-8 Assay

Human ccRCC cell line 786-O and human renal cell adenocarcinoma cell line ACHN were obtained from the Cell Bank of the Chinese Academy of Sciences (Shanghai, China). The cells were cultured in Roswell Park Memorial Institute (RPMI) 1,640 supplemented with 10% fetal bovine serum (FBS) in an environment of 5% CO_2_ and 37°C and then seeded onto the six-well plates. After the confluence of 50–60% was reached, they were induced with a medium containing sunitinib for 48 h and then exposed to a fresh medium without sunitinib for 24 h. Cells that stably proliferate were then exposed to a 1 μM higher concentration than the previous, and the treatment ended when the cells exhibited normal viability under the targeting sunitinib concentration of 8 μM (Sakai et al., 2013; Peng et al., 2019). Then, they were used as sunitinib-resistant cell lines for the following research. The IC50 values for 786-O/786-OR were 4.15 μM/11.16 μM and 3.72 μM/9.52 μM for ACHN/ACHNR, respectively.

To detect the viability of cells, 5 × 10^3^ cells were seeded onto 96-well plates and treated with a medium with 10% FBS for 24 h. Different concentrations of sunitinib were added into different wells, and the cells were treated for 48 h. Ten microliters of Cell Counting Kit-8 (CCK8) reagent (Beyotime Biotechnology, Shanghai, China) was added into the wells, and the cells were incubated in an environment of 37°C and 5% CO_2_ for 2 h. At last, the OD450 was detected using an Epoch Microplate Spectrophotometer (BioTek Instruments, Inc., Winooski, VT, United States), and the cell viability and inhibition rate could be calculated.

### Quantitative Real-Time PCR and Western Blotting

Total RNA was isolated from two cell lines by TRIzol Reagent (Thermo Fisher Scientific, Waltham, MA, United States) and reversely transcribed into cDNAs using the reverse transcription kit (Thermo Fisher Scientific, United States) following the manufacturer’s protocol. Then, as the manufacturer’s protocol suggests, the qRT-PCR was conducted on a LightCycler 480 II (Roche Diagnostics, Basel, Switzerland) instrument by using the SYBR-Green master kit (Vazyme, Nanjing, China). The primers used to amplify MX2 were purchased from Invitrogen (Shanghai, China), which were designed as 5′-TGAACGTGCAGCGAGCTT-3′ (forward) and 5′-GGCTT GTGGGCCTTAGACAT-3′ (reverse). The primers for β-actin were 5′-CCCATCTATGAGGGTTACGC-3′ (forward) and 5′-TTTAATGTCACGCACGATTTC-3′ (reverse). Each qRT−PCR was performed in triplicate, and β-actin was utilized as a control to normalize MX2 expression.

Total proteins were extracted from cells using radioimmunoprecipitation assay (RIPA) buffer, which contains protease inhibitors. BCA Protein Assay kit (Beyotime Biotechnology, Shanghai, China) was used to determine the protein concentration. Proteins were separated by 10% sodium dodecyl sulfate–polyacrylamide gel electrophoresis (SDS-PAGE) and transferred to a polyvinylidene difluoride (PVDF) membrane. After being blocked within skim milk for 2 h, the membranes were incubated overnight with the primary antibody specifically against MX2 at 4°C. After that, membranes were incubated with the secondary antibody anti-mouse IgG (Cell Signaling Technology, Danvers, MA, United States) for 2 h. GAPDH expression was used as a loading control. Protein bands were visualized with an enhanced chemiluminescence (ECL) detection system (Thermo Fisher Scientific, Rochester, NY, United States).

### RNA Interference and Colony Formation Assay

RNA interference of MX2 was accomplished using small interfering RNA (siRNA). 786-OR cells were transfected with siRNA-MX2 using Lipofectamine 3,000 (Invitrogen). qPCR was used to evaluate the efficiency of siRNA interference. The MX2 knockdown cells were then seeded onto 30-mm cell culture dishes containing 10% FBS. Sunitinib with a concentration of 8 μM was added into the dishes, and the cells were cultured for 14 days. The medium was changed every 3 days. After that, the cells were fixed with 4% formaldehyde for 15 min and stained with 0.1% crystal violet for 20 min before counting the number of colonies.

### Statistical Analysis

All analyses were performed using R version 3.6.1, SPSS version 23.0, GraphPad Prism 9.0, and ImageJ software. All statistical tests were two-sided, and a *p*-value less than 0.05 was considered statistically significant.

## Results

### Identification of Differentially Expressed Genes Related to Sunitinib Resistance in Clear Cell Renal Cell Carcinoma

**FIGURE 1:**
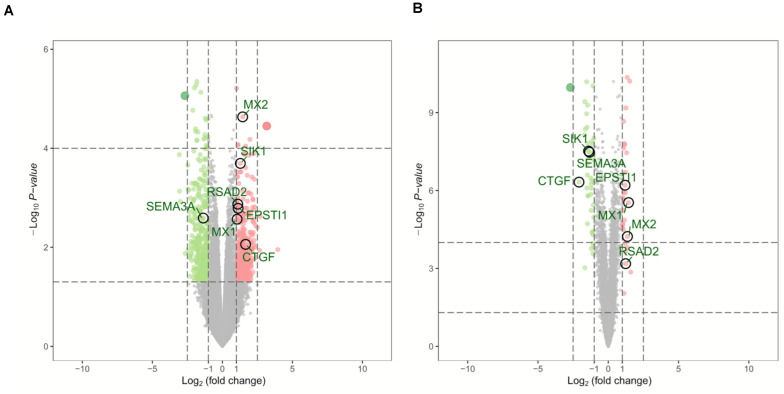
Volcano plots of DEGs in GSE64052 and GSE76068. **(A)** DEGs in the GSE64052 dataset. **(B)** DEGs in the GSE76068 dataset. Red dots represent upregulated genes, and green dots represent downregulated genes. DEGs, differentially expressed genes.

Two microarray datasets (GSE64052 and GSE76068) from the GEO database were used to analyze gene expression of ccRCC samples either sensitive or resistant to sunitinib. The limma package was used to normalize the data (| log2FC| > 1 and corrected *p* < 0.05). Subsequently, 690 DEGs were obtained from GSE64052, including 365 down- and 325 upregulated genes; 70 DEGs were obtained from GSE76068, including 42 down- and 28 upregulated genes. As a result, a total of 760 DEGs associated with sunitinib resistance in ccRCC were identified. Volcano plots were used to visualize the DEGs, as is shown in Figure 1.

### Gene Set Enrichment Analysis of Differentially Expressed Genes Related to Sunitinib Resistance in Clear Cell Renal Cell Carcinoma

**FIGURE 2:**
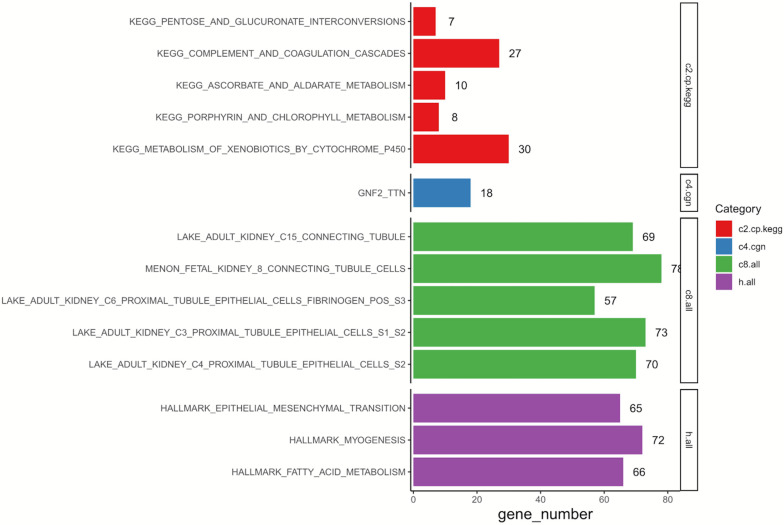
Results of GSEA on 760 DEGs. C2, curated gene sets; C4, computational gene sets; C8, cell type signature gene sets; Hallmark, hallmark gene sets; GSEA, gene set enrichment analysis; DEGs, differentially expressed genes; KEGG, Kyoto Encyclopedia of Genes and Genomes.

GSEA was conducted to annotate the potential biological role of DEGs in resistant ccRCC. As is shown in Figure 2, by setting *p-*value < 0.05 as the cutoff criteria, up to 650 genes among 760 DEGs were enriched. The results indicated that the representative Kyoto Encyclopedia of Genes and Genomes (KEGG) pathway was “metabolism of xenobiotics by cytochrome p450,” and these DEGs were tightly related to “proximal/connecting tubules epithelial cells” in adults. Three main associated biological processes were “epithelial—mesenchymal transition” (EMT), “myogenesis,” and “fatty acid metabolism.”

### Identification of Hub Genes

**FIGURE 3:**
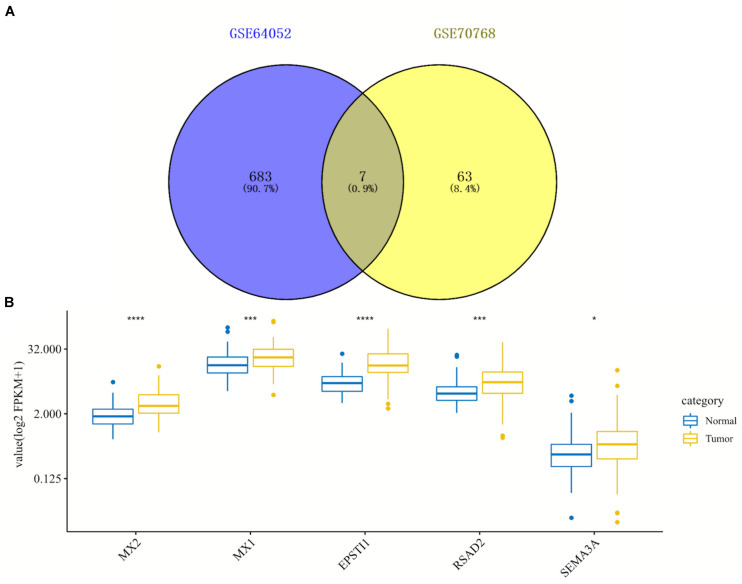
Identification and expression analysis of five hub genes in normal/tumoral tissues. **(A)** Venn diagram of seven overlapping genes by comparing differentially expressed genes (DEGs) from two datasets. **(B)** Box plot of expression of five hub genes in normal and tumoral tissues in TCGA database. DEGs, differentially expressed genes; TCGA, The Cancer Genome Atlas. *p < 0.05; ***p < 0.001; ****p < 0.0001.

By comparing two datasets (GSE64052 and GSE76068), seven overlapping DEGs were obtained including MX2, MX1, SIK1, EPSTI1, RSAD2, SEMA3A, and CTGF, as is shown in the Venn diagram (Figure 3A). Under the inclusion criteria of being concurrently upregulated or downregulated in two datasets, five hub genes were finally identified: MX2, MX1, EPSTI1, RSAD2, and SEMA3A. Then we detected the expression of these five genes in 72 paired normal and tumoral kidney tissues in TCGA database, as shown in the box plot (Figure 3B). All hub genes showed an increase in expression to different degrees in tumors compared with normal tissues.

### Kaplan–Meier Curves and Protein–Protein Interaction Network of Hub Genes

**FIGURE 4:**
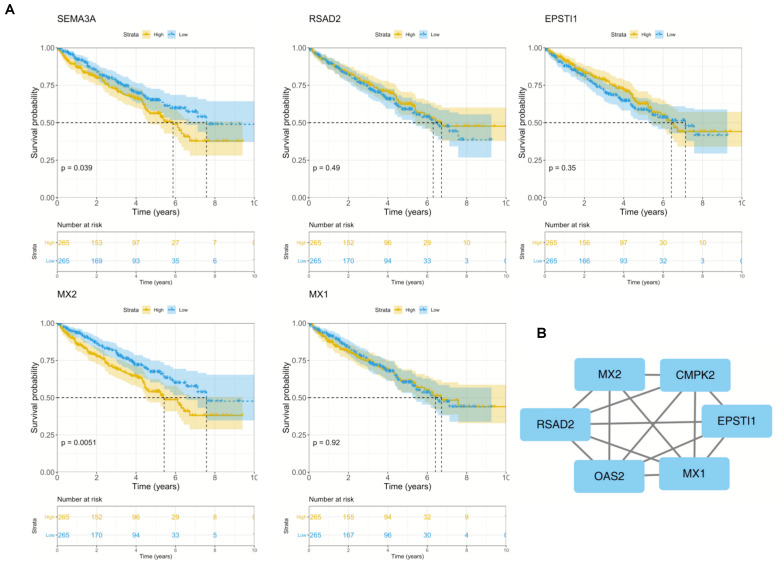
Survival analysis and PPI network of five hub genes. **(A)** Kaplan–Meier curves of five hub genes in TCGA cohort. **(B)** Representative PPI network of hub genes. PPI, protein–protein interaction; TCGA, The Cancer Genome Atlas.

To further explore the prognostic role of hub genes in sunitinib-resistant ccRCC patients, survival analysis was conducted by R software according to the clinical information in TCGA database. Among five hub genes, high levels of MX2 (*p* = 0.0051) and SEMA3A (*p* = 0.039) were linked with shorter OS in sunitinib-resistant patients as compared with low levels, while the rest of them showed no differences (Figure 4A). The STRING was used to construct the PPI network of all DEGs. Then, the most significant module was recognized by Cytoscape, which included four of them (Figure 4B).

### Key Gene MX2 Promotes the Malignant Phenotype of Clear Cell Renal Cell Carcinoma

**FIGURE 5:**
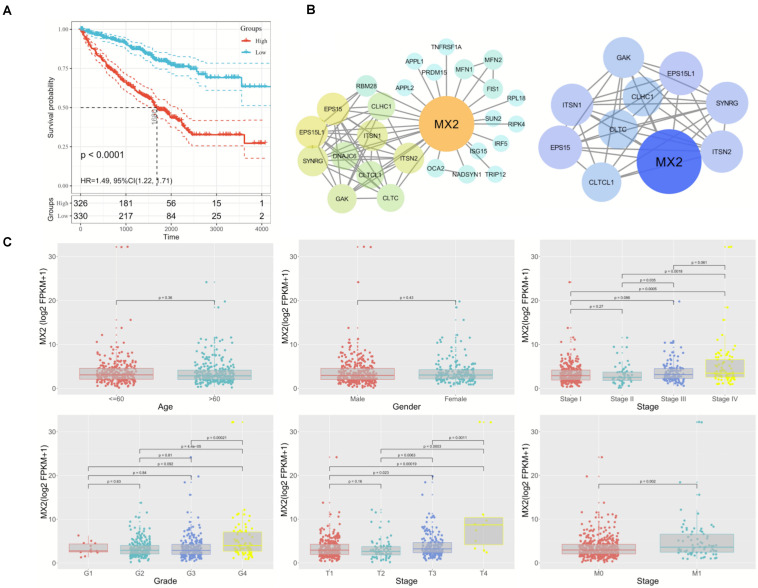
Prominent clinical correlation of MX2. **(A)** Results of survival analysis on patients derived from KIRC, ICGC, and GSE29609. **(B)** PPI network of MX2 and top 10 nodes by STRING and Cytoscape. **(C)** The mRNA levels of MX2 in patients from TCGA database with various clinicopathological characteristics: gender, age, grade, T stage, M stage, and stage. Data differences were tested by one-way ANOVA. TCGA, The Cancer Genome Atlas; ICGC, International Cancer Genome Consortium.

Considering the strong association between patient prognosis and MX2 expression, MX2 was chosen as the candidate gene for the following research. In addition to the KIRC database, complete survival information of patients from the ICGC and GSE29609 was added for more comprehensive survival analysis. The result showed higher MX2 level is significantly related to poorer prognosis in ccRCC patients (*p* < 0.001, Figure 5A). MX2-based PPI network revealed the potential interacting proteins (Figure 5B). Then, the results of clinical correlation analysis indicated that MX2 expression exhibited a prominent increase in T4 stage compared with T1 stage (*p* < 0.001), in M1 stage compared with M0 stage (*p* = 0.002), and in stage 4 compared with stage 1 (*p* < 0.001). The clinicopathological information of 539 ccRCC patients from TCGA database and their association with MX2 level are shown in Table 1. These findings may establish the crucial role of MX2 in promoting the malignancy of ccRCC.

**TABLE 1:** Correlation between MX2 level and clinicopathological parameters of ccRCC patients.

Characteristics	MX2 expression		Total	*P*-value
	Low	High		
Total cases	265	265	530	
Age				
<60	120	124	244	ns
≥60	145	141	286	
Gender				
Male	175	170	345	ns
Female	90	95	185	
G Grade				
I-II	126	114	240	ns
III-IV	133	149	282	
Stage**				
I-II	189	156	345	0.0026
III-IV	76	109	185	
T stage*				
I-II	182	157	339	0.0237
III-IV	83	108	191	
Metastasis*				
No	235	216	238	0.0335
Yes	19	33	41	

Chi-square test or Fisher’s exact test was used for statistical analysis, *p < 0.05, **p < 0.01.

### Gene Set Variation Analysis on MX2

**FIGURE 6:**
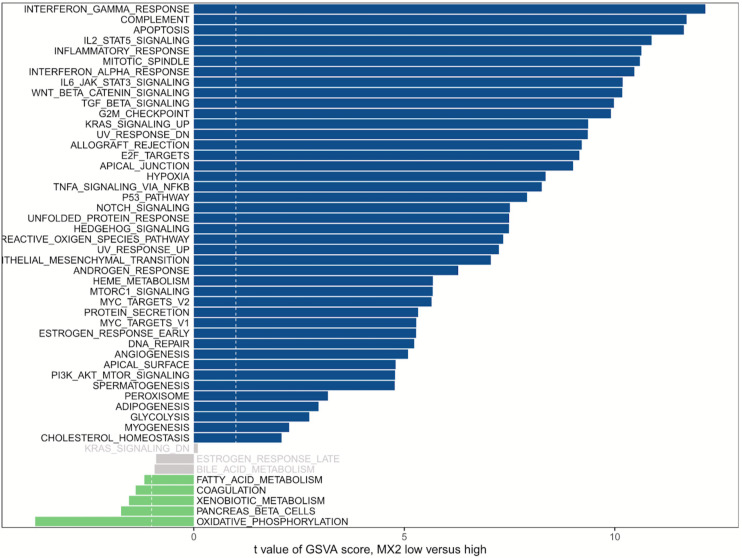
GSVA of MX2. MX2 is involved in certain pathways contributing to antiangiogenic therapy resistance. Blue bars represent high MX2 expression, and green bars represent low MX2 expression. GSVA, Gene Set Variation Analysis.

GSVA was used to detect pathway differentiations over a cohort sample derived from TCGA. After *p* < 0.05 was set as the cutoff criteria, 42 upregulated classic pathways were identified in the high MX2 group. The plot illustrated that MX2 participated in several pathways relevant to antiangiogenic therapy resistance, and representative pathways included “Hypoxia,” “IL-6–JAK–STAT3 signaling,” “TGF-β signaling,” and “PI3K–AKT–mTOR signaling” (Figure 6).

### MX2 Expression in Sunitinib-Resistant Clear Cell Renal Cell Carcinoma Cell Lines

**FIGURE 7:**
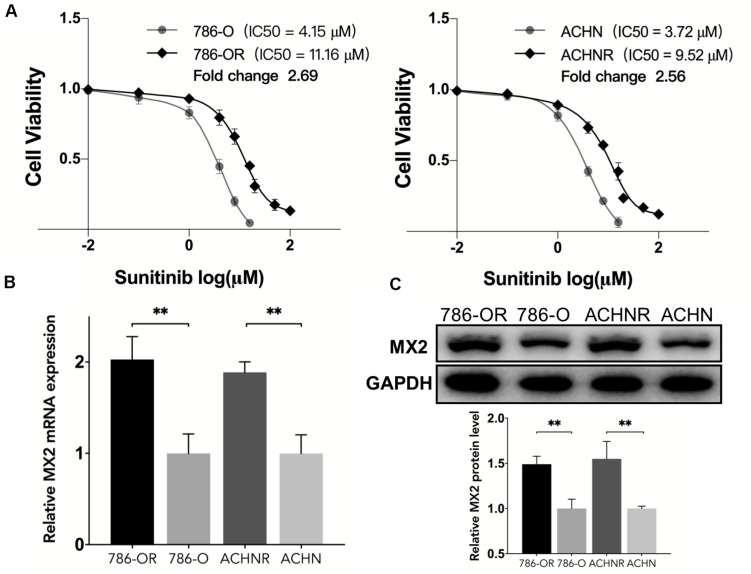
Upregulation of MX2 expression in sunitinib-resistant cell lines. **(A)** CCK8 assay indicated the formation of sunitinib resistance in 786-O and ACHN cells. **(B)** Relative mRNA expression of MX2 by qPCR. **(C)** Relative protein expression of MX2 by Western blotting. The error bars represent mean ± SD. Data differences were tested with Student’s *t*-test (^∗∗^*p* < 0.01).

To verify the findings obtained through bioinformatics analysis, two sunitinib-resistant RCC cell lines (786-OR and ACHNR) were established by incubating the cells with sunitinib as mentioned before. The results of the CCK8 assay indicated that 786-OR and ACHNR showed much higher cell viability than the parental cells (Figure 7A). The fold change of IC50 values verified the formation of resistance to sunitinib in two cell lines. Then we detected MX2 expression by conducting qRT-PCR and Western blotting (WB) in 786-O/786-OR and ACHN/ACHNR, to find that both mRNA and protein levels exhibited an increase in resistant cell lines (Figures 7B,C).

### MX2 Knockdown Re-sensitize the Cells to Sunitinib Through PTEN/Akt Signaling

**FIGURE 8:**
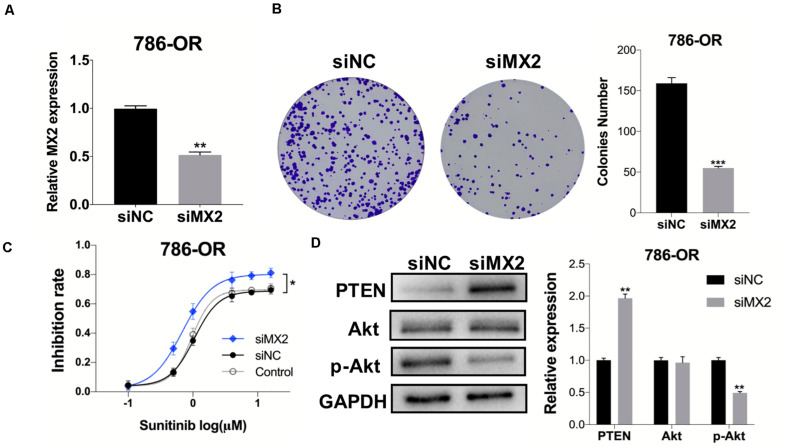
MX2 knockdown sensitized cells to sunitinib through PTEN/Akt signaling. **(A)** Relative MX2 mRNA expression in 786-OR cells after being transfected with siRNA. **(B)** Results of colony formation assay of 786-OR after being transfected with siRNA. **(C)** Result of inhibition rate assay on 786-OR cells after transfected with siRNA. **(D)** Relative protein level of PTEN, Akt and p-Akt in 786-OR cells after transfected with siRNA. The error bars represent mean ± SD. Data differences were tested with Student’s *t*-test (*p < 0.05; **p < 0.01; ***p < 0.001).

After the validation of MX2 expression in resistant cell lines was acquired, the 786-OR cells transfected with siRNA-MX2 were selected to conduct the following mechanism studies. Based on the result of qPCR, which proved the efficiency of transfection (Figure 8A), the colony formation assay was conducted, and the knockdown of MX2 strongly attenuates the proliferation capability of resistant cells when exposed to sunitinib (Figure 8B). MX2 knockdown also sensitized 786-OR cells to the cytotoxic effect of sunitinib as shown in the inhibition rate assay (Figure 8C). Finally, the expression of PTEN/Akt signaling was detected by WB. The results showed that MX2 knockdown upregulated the PTEN level while downregulating the p-Akt expression in 786-OR cells (Figure 8D). These results may reveal the potential mechanisms of how MX2 promotes sunitinib resistance in ccRCC.

## Discussion

From conventional immunotherapy (represented by cytokines) to small molecular targeted agents (represented by multi-targeted TKIs and mTOR-targeted agents), tremendous changes have happened to RCC therapeutic standards. Despite the robust effects of targeted agents on treating mRCC patients, many patients fail to show responses after a median time of 6–15 months of treatment (Molina et al., 2014) and finally no longer benefit from this kind of therapy. Rini et al. offered comprehensive insights into the mechanisms of resistance to targeted therapy by reviewing the latest studies and clinical data in this field (Rini and Atkins, 2009). Various hypotheses were successively raised, including angiogenic escape (Schor-Bardach et al., 2009), revascularization (Bazelaire et al., 2008), tumor hypoxia-driven upregulation of HIF1A, and alternative proteins or pathways (Fischer et al., 2007). Notably, targeting angiogenesis itself may encourage tumor invasiveness, due to the EMT of cells aimed to escape the hypoxic microenvironment caused by lessened vascularization (Bhullar et al., 2018).

Recently, a growing number of studies have been noted that focus on the key molecules and potential pathways involved in sunitinib resistance in ccRCC. Huang and colleagues found that ccRCC tumors can be re-sensitized to sunitinib treatment with the coadministration of an IL-8 neutralizing antibody and confirmed that IL-8 is a potent contributor to sunitinib resistance in ccRCC (Huang et al., 2010). Adelaiye-Ogala et al. (2017) found that modulating EZH2 activity suppressed phosphorylation of certain RTKs, thus restoring the antitumor effects of sunitinib acquired or intrinsically resistant ccRCC. By uncovering the specific molecular mechanisms, Lu et al. (2019) and Xiao et al. (2017) demonstrated the promotive role of miR-15b and miR-144-3p in regulating resistance to sunitinib in RCC, respectively. From the perspective of tumor immunology, increased infiltration of CD4/8^+^ T cells (Liu et al., 2015) and TNF-α (Mikami et al., 2015) was identified in antiangiogenic therapy-resistant RCC primary tumors and was related to worse OS in patients. These findings indicated the participation of tumor immune microenvironment in antiangiogenic TKIs resistance in RCC.

In our study, we obtained 760 DEGs by comparing two datasets from the GEO database. According to the results of GSEA on DEGs, these genes were enriched in several antidrug resistance-related pathways and biological processes in ccRCC. For instance, the representative KEGG pathway was the metabolism of xenobiotics by cytochrome p 450. Taking tamoxifen and imatinib for example, Rochat highlighted the indispensable role of cytochrome p 450 isoenzymes (CYPs) in anticancer drug resistance and safety by mediating the drug metabolism (Rochat, 2005). Simultaneously, sunitinib also increased the levels of CYP1A1 mRNA and protein through AhR ligand-dependent mechanisms in MCF7 cells, which was proved for the first time by Maayah et al. (2013).

In the present study, we mainly introduced a novel molecule MX2 and its role in sunitinib resistance during ccRCC treatment. The outcomes of survival analyses suggested MX2 may serve as an indicator for the prognosis of sunitinib-resistant ccRCC patients. The GSVA then uncovers the potential function of MX2 in the TKIs therapeutic response by revealing some pathways that MX2 could be involved in. Then, the qPCR and WB were conducted and successfully validated the aberrant upregulation of MX2 in resistant cells. According to the results of colony formation and inhibition rate assay, the 786-OR cells were sensitive again to the sunitinib after being transfected with siRNA targeting MX2. These results may establish the promotive role of MX2 in the formation of resistance to sunitinib in ccRCC patients. To understand the mechanism of how MX2 links to sunitinib resistance, expression of PTEN/Akt signaling was detected by WB, and it turned out that MX2 level was inversely correlated with the PTEN level while consistent with the p-Akt level in cells. Many previous studies have reported that the PTEN/PI3 k/Akt signaling was involved in EGFR-TKI resistance in the non-small cell lung cancer (NSCLC) (Fang et al., 2012; Pérez-Ramírez et al., 2015; Wang et al., 2018; Choi et al., 2019). This signaling was also found as the driver of drug resistance in breast cancer (Araki and Miyoshi, 2018) and hepatocellular carcinoma (Akula et al., 2013). Hafsi et al. (2012) then summarized the crucial role of PI3 k/PTEN/Akt pathway in the formation of drug resistance due to its role of regulating cell growth. What is more, Sekino et al. (2020) proved that knocking out PTEN could decrease the sensitivity to both sunitinib and sorafenib in RCC cells, which indicated the active participation of PTEN-related pathway in regulating cell responses to targeted agents. These findings were consistent with our result in the present study.

MX2, a common human myxovirus resistance gene, encodes MXB protein, which belongs to both the dynamin family and the GTPase family. It is well established that MX dynamin-like GTPases (MXA and MXB) are pivotal antiviral effector proteins of the IFN system, working by blocking the early steps of the replication cycle to inhibit several viruses (Haller et al., 2015). Nevertheless, in comparison with mature studies on the HIV-1 restriction function and antiviral effect of MX2, seldom do studies discuss its role in cancers. Until recently, Choi and colleagues found the imperative role of MX2 in melanoma susceptibility through an integrative approach (Choi et al., 2020). Juraleviciute et al. (2020) confirmed that MX2 downregulation promoted melanoma proliferation, as well as a high level of MX2 was linked to better patient survival, which proved that MX2 was a tumor suppressor gene by regulating the cell cycle in melanoma. However, its functions in antiangiogenic targeted therapies and tumorigenesis of ccRCC have never been studied. In the present study, we found that MX2 level was tightly related to the prognosis of ccRCC patients. The clinical correlation analysis also showed its strong association with the malignant phenotype of ccRCC. These findings may add to the research on the biological role of MX2 in genitourinary cancers as a supplement, particularly in renal carcinoma.

The limitations of this study are obvious. First, it lacked enough gene expression data of ccRCC samples because we only obtained 13 paired samples either sensitive or resistant to sunitinib from two datasets. Second, in the construction of sunitinib resistant cell lines, an increased sunitinib concentration of roughly 1 μM higher each time may limit the tolerance of cells. Besides, the final concentration did not meet the requirements of up to 10 μM or higher (Sakai et al., 2013), which could probably cover up the potential larger distinction of MX2 expression between sensitive and resistant cells. Most importantly, further experiments are urgently needed to elucidate the detailed mechanisms of how MX2 affects the efficiency of sunitinib.

Taken together, our work offers new insights into the understanding of sunitinib resistance in ccRCC. We believe that with further investigation, promising therapeutic approaches to overcome the dilemma of antiangiogenic therapies in RCC would come soon.

## Conclusion

In ccRCC, MX2 is upregulated in the sunitinib-resistant group compared with the sensitive group, as well as in tumors compared with normal tissues. High-level MX2 is significantly correlated with shorter OS, higher clinical stage, and poor prognosis in metastatic ccRCC patients. Moreover, MX2 could decrease the sensitivity of tumor cells to sunitinib probably through PTEN/Akt pathway. In conclusion, our study suggests MX2 a potent indicator for resistance to sunitinib and a therapeutic target in ccRCC patients.

## Data Availability Statement

The original contributions presented in the study are included in the article/Supplementary Material, further inquiries can be directed to the corresponding author/s.

## Ethics Statement

Written informed consent was obtained from the individual(s) for the publication of any potentially identifiable images or data included in this article.

## Author Contributions

YW and PS designed the study. XR collected and analyzed the data. XC and BW finished the experiments. QZ, HB, and JQ sourced the literature. YW edited the manuscript. PS provided the funding and supervised the whole study. All authors contributed to the article and approved the submitted version.

## Conflict of Interest

The authors declare that the research was conducted in the absence of any commercial or financial relationships that could be construed as a potential conflict of interest.

## Footnotes

1 http://string-db.org

## Abbreviations

RCC: renal cell carcinoma
ccRCC: clear cell renal cell carcinoma
VEGF: vascular endothelial growth factor
VEGFR: vascular endothelial growth factor receptor
mTOR: mammalian target of rapamycin
VHL: von Hippel-Lindau
TKIs: tyrosine kinase inhibitors
DEG: differentially expressed genes
GEO: gene expression omnibus
GSEA: gene set enrichment analysis
PPI: protein—protein interaction
OS: overall survival
GSVA: Gene Set Variation Analysis
MX2: Human myxovirus resistance protein 2
TCGA: The Cancer Genome Atlas
FBS: fetal bovine serum
KEGG: Kyoto Encyclopedia of Genes and Genomes
ICGC: International Cancer Genome Consortium.
